# Prevalence and potentially prognostic value of C‐circles associated with alternative lengthening of telomeres in canine appendicular osteosarcoma

**DOI:** 10.1111/vco.12665

**Published:** 2020-12-08

**Authors:** Ludmila Bicanova, Theresa Kreilmeier‐Berger, Martin Reifinger, Klaus Holzmann, Miriam Kleiter

**Affiliations:** ^1^ Department for Companion Animals and Horses University of Veterinary Medicine Vienna Austria; ^2^ Department of Pathobiology University of Veterinary Medicine Vienna Austria; ^3^ Department of Medicine I, Division: Institute of Cancer Research, Comprehensive Cancer Center Medical University of Vienna Vienna Austria

**Keywords:** C‐circle assay, comparative oncology, human and dog, osteosarcoma, prognosis, telomere maintenance mechanism

## Abstract

Alternative lengthening of telomeres (ALT) is a telomerase‐independent telomere maintenance mechanism (TMM) with high prevalence in human osteosarcomas but remains unknown in canine osteosarcomas. The aim of this study was to evaluate the prevalence of ALT by detection of extra‐chromosomal circles of telomeric DNA and to assess clinical outcome in canine patients with spontaneous occurring appendicular osteosarcoma. Fifty dogs with histopathological confirmed osteosarcomas were included into this study. Medical records were retrospectively analysed for patient characteristics, oncologic therapy and survival. DNA was isolated from archived FFPE tumour tissue specimens and applied for C‐ and G‐circle assay (CCA and GCA) and for telomeric content (TC) measurement with radiolabeled probes. ALT activity was detected for 10 of 50 (20%) cases by CCA. Four CCA positive cases were detected even with input DNA below 1 ng and demonstrated the high sensitivity of CCA for canine tumours. G‐circles and TC were not suitable to distinguish CCA positive and negative cases. CCA‐status showed an association with male gender and Rottweiler breed. Dogs with CCA positive osteosarcomas had shorter overall survival times than patients with CCA‐tumours and CCA‐status was a significant prognostic factor besides treatment in the Cox proportional hazard model. These findings make canine osteosarcomas an interesting model for comparative TMM research, but future studies are warranted to investigate if CCA‐status can serve as novel prognostic marker.

## 1 INTRODUCTION

Telomeres are conserved nucleoprotein complexes that cap both ends of each chromosome to protect the cell from DNA damage response.^1^ Mammals, including humans and dogs, use telomere sequences of tandem DNA hexanucleotide repeats (TTAGGG)n in a multiplicity length.^2^, ^3^ During the life span of normal somatic cells, telomeres shorten progressively and lead to replicative senescence or apoptotic cell death.^1^ In cancer cells, activation of a telomere maintenance mechanism (TMM) is an essential step for immortalization.^4^ In cancer, there are two known TMMs, telomerase activity (TA) and alternative lengthening of telomeres (ALT). The majority of human and canine cancer types activate the enzyme telomerase, as TMM,^3^, ^5^ whereas 4% to 11% of human tumours use the ALT‐pathway.^6^ In a recent large‐scale study across 31 human cancer types telomerase was activated in 73%, ALT in 5% and no TMM activation was observed in 22%.^7^, ^8^, ^9^ Comparable to humans, several studies reported that more than 90% of canine tumours utilize telomerase as TMM.^10^, ^11^, ^12^, ^13^ In contrast, information about ALT in canine tumours is currently limited.^14^

ALT is a telomerase‐independent TMM based on homologous recombination with potential as a quantifiable clinical marker and as a target for cancer therapy.^6^, ^15^ For the detection of ALT certain characteristics have been used in humans including long and heterogeneous telomere length, colocalization of telomeres with ALT‐associated PML bodies, mutation/loss of ATRX/DAXX or presence of C‐circles.^16^, ^17^, ^18^, ^19^ In dogs, ALT‐associated characteristics, such as long heterogeneous telomeres, overexpression of p53, colocalization of DAXX with telomeres and presence of C‐circles, have been described.^14^

C‐circles are extra‐chromosomal circles of telomeric DNA that are specific for ALT activity.^15^ The detection of C‐circles has developed to a front‐line assay for identification of ALT activity.^16^, ^19^, ^20^ The C‐circle assay (CCA) involves extraction of DNA, amplification of self‐primed C‐circles with rolling circle amplification (RCA) and detection of the amplification products by native telomeric dot blot or telomeric qPCR.^19^ Besides C‐circles, G‐circles are ALT specific and have the ability to self‐prime RCA of telomeric G‐strand templates, but they are about 100‐fold less abundant.^15^, ^16^ The CCA is the first ALT assay that has a quantitative correlation with ALT activity levels and can be used for liquid biopsies as well as in FFPE archived human and canine tissue.^14^, ^15^, ^21^

Certain tumour types seem to utilize ALT more frequently. Especially, in tumours originating from mesenchymal tissues, such as sarcomas, the ALT phenotype is overrepresented.^7^, ^8^, ^9^ Sarcomas are a heterogeneous cancer group and ALT status has been demonstrated as negative prognostic factor in some sarcoma subtypes.^22^, ^23^, ^24^, ^25^ In dogs, the ALT mechanism has been identified in various sarcoma subtypes and as in humans different sarcoma subtypes seem to have a heterogeneous ALT prevalence.^14^ In human osteosarcomas, high ALT prevalence rates from 47% to 80% are observed.^26^, ^27^, ^28^, ^29^ Osteosarcoma (OSA) is a highly malignant bone tumour in humans and dogs, characterized by a high metastatic rate and low overall survival rates. In both species, it represents the most common primary malignant skeletal neoplasia.^4^, ^30^, ^31^, ^32^ The annual incidence in people is reported with approximately one to five cases per million population. In comparison to humans, OSA incidence rates in dogs are about 27 times higher.^32^, ^33^, ^34^ This high incidence rate would make canine osteosarcomas an interesting model for comparative TMM research. However, the clinical relevance of ALT in canine osteosarcomas is currently unknown. Our previous study in various sarcomas included four osteosarcomas and one showed ALT activity.^14^

Thus, the aim of this study was to evaluate the prevalence of ALT in a larger cohort of canine appendicular osteosarcomas together with clinical outcome.

## 2 MATERIALS AND METHODS

### 2.1 Patient cohort and clinical data

Dogs with available archived formalin‐fixed and paraffin embedded (FFPE) appendicular osteosarcoma samples that had been collected during routine diagnostic work‐up and therapy at our institution between 2003 and 2016 were included into this study. Medical records were analysed for the following information: Patient signalment, tumour location and tumour subtype, disease stage, pretreatment alkaline phosphatase (ALP) level, therapy and outcome. Routine staging procedures included radiographs of the primary lesion, three‐view thoracic radiographs, CBC and biochemistry profile. In patients with hind limb lesions an abdominal ultrasound was performed. Additional staging procedures were only obtained if clinical findings or routine staging results required further diagnostic work‐up. Dogs were assigned to the age categories “young” (<5 years), “middle‐aged (5‐10 years) and “old (>10 years). Further, patients were assigned to the three size categories “small‐medium” (< 30 kg), “large” (30‐60 kg) and “giant” (> 60 kg). Median overall survival time (OST) was defined as the time from diagnosis until death.

### 2.2 Spontaneous OSA tissue samples and controls

Fifty archived FFPE tumour tissue samples from spontaneous occurring therapy naïve appendicular OSAs were analysed for the presence of ALT activity. Additionally, five already known canine sarcoma tissue samples with ALT activity as well as human ALT cell lines U2OS from osteosarcoma and YT‐BO from astrocytoma served as positive controls.^14^, ^35^ Two human cell lines with telomerase activity served as negative controls. These cell lines included SW480 derived from a colon adenocarcinoma and the cell line T98G (CRL‐1690) derived from a glioblastoma multiforme. Origin and handling of these cell lines was recently described.^14^

Cell line validation statement: All cell lines except YT‐BO are validated ATCC cell lines. Additionally, in all cell lines except U2OS cell line authentication testing was performed.

### 2.3 DNA extraction and quantification

DNA from five to ten 10 μm thick sections of FFPE archived tumour tissue samples was extracted using nexttec 1‐Step DNA Isolation Kit for Tissue and Cells (nexttec Biotechnology GmbH, Hilgertshausen‐Tandern, Germany). After dewaxing with xylene and cell lysis overnight, DNA was extracted.^14^ DNA isolation from human control cell lines was performed with Quick C‐Circle Preparation (QCP) protocol.^19^

A volume of 2 μL corresponding to 1/50 of nexttec and 1/25 of QCP extracted DNA were quantified with Qubit fluorometer (Invitrogen, Carlsbad, CA) using iQuant BR and HS dsDNA Quantification Kits (GeneCopoeia, Rockville, MD, USA) for detection of dsDNA between 0.2 and 1000 ng. Therefore, a minimum of 5 ng or 0.1 ng/μL extracted DNA could be measured. DNA samples were stored at −80°C.

### 2.4 Radiolabel C‐ and G‐circle assay and telomeric content

The presence of ALT activity was assessed using the radiolabel CCA method.^19^ In detail, in a first step, extracted DNA was diluted to 32 ng/μL (dilution 1). For this, 2 μL of pure DNA extract were diluted in an appropriated amount of 10 mM Tris HCl pH 7.6 buffer for FFPE tissue samples or QCP buffer for human control cell lines. QCP sample dilution 1 was performed with QCP buffer to standardize total amount of QCP buffer used in the CCA reaction for all samples. This process is necessary to minimize inhibitory effects of QCP lysis buffer.^19^ Then 2.5 μL of dilution 1 was further diluted 1:1 with 2.5 μL Tris buffer (dilution 2). A volume of 2 μL of dilution 2 were finally mixed with 8 μL Tris buffer to a total volume of 10 μL (dilution 3). If the DNA quantity was below 32 ng/μl, dilutions 1 and 2 were skipped over and up to 4 μL of pure DNA extract were mixed with Tris buffer to a total volume of 10 μL to reach DNA quantities closest to 32 ng input DNA. For QCP isolated DNA dilutions 1 and 2 could always be performed. Then 10 μL of dilution 3 was mixed with 10 μL Master Mix including 7.5 units phi29 DNA polymerase (New England Biolabs Inc., Ipswich, MA, USA) for phi+ CCA reactions, or with Master mix including same aliquote ddH2O as replacement for the enzyme for phi‐ CCA reactions used as control. Rolling circle amplification (RCA) of C‐circles (CC) was performed at 30°C for 8 hours on a peqSTAR PCR cycler (Peqlab Biotechnologie GmbH, Erlangen, Germany). Polymerase was inactivated at 70°C for 20 minutes and CCA reactions were kept overnight at 4°C and/or frozen at −20°C. For C‐circle (CC) detection, 20 μL CCA products were diluted with 40 μL 2× saline‐sodium citrate (SSC) to a total volume of 60 μL and loaded each into 96‐well positions of a Bio Dot Apparatus (Bio‐Rad Laboratorities Inc., Hercules, CA, USA). DNA was transferred by vacuum on 0.45 μm Biodyne B Membran (cat# P/N 60208, Pall Corporation Life Science, Port Washington, NY, USA). Dried dot blot membranes were irradiated twice with UV light applying 1200μJoule *1000 (UV Stratalinker 1800, Stratagene, San Diego, CA, USA) to crosslink DNA.

Oligonucleotides were synthesized by Eurofines (Germany). Published sequences were used for ctel and alu oligonucleotides,^19^ for gtel oligonucleotide sequence was 5′‐TTAGGGTTAGGGTTAGGG‐3′.^36^, ^37^ For each labelling reaction 1 μL containing 100 pmol oligonucleotide were mixed with 10 μL ddH_2_O and 2 μL polynucleotide kinase (PNK) buffer and incubated at 65°C for 2 minutes and put on ice. Then 2 μL (10 U/μL) of Ambion cloned T4 PNK (cat# AM2310, InvitrogenThermofisher, MA, USA) and 5 μL (50 μCi) EasyTide ATP‐gamma‐32P with specific activity of 6000 Ci (222TBq)/mmol (cat# NEG502Z250UC, Perkin Elmer, Waltham, MA, USA) were added, mixed and incubated 30 minutes at 37°C. Radiolabeled oligonucleotides were purified by Biosciences ProbeQuant G‐50 Micro Columns (#27‐5335‐01, Amersham Biosciences, Little Chalfont, UK) and incorporation rates were determined using a scintillation counter. Dot blot membranes with dimension 9 × 12cm were used for hybridizations in series, starting with radiolabeled oligonucleotide ctel, followed by alu and gtel. Membranes were mounted in tubes (l × d = 150 × 35mm) and placed in Hybaid Shake 'n' Stack hybridization oven (Thermo Electron, Waltham, MA, USA) with rotation as recommended by the manufacturer. Dot blot membranes were incubated at 37°C for at least 30 minutes with 7 mL PerfectHyb Plus Hybridization Buffer (cat# H7033, Sigma, Kawasaki, Japan) for prehybridization and overnight with 7 mL of fresh Hybridization Buffer including 10% to 20% of radiolabeled oligonucleotides with activity of 0.6‐1 × 10^5^ cpm/mL similar as previously published with 5 × 10^5^ cpm/mL.^19^ Afterwards blots were washed three times with 0.5×SSC/0.2% SDS at 37°C. Membranes were dried, mounted on paper and radiolabel signals were measured for ctel and gtel overnight and for alu over weekend using Storage Phosphor Screens (Amersham Biosciences, Little Chalfont, UK) and Typhoon scanner (GE Healthcare, Chicago, IL, USA). Signals were removed from blots by washing three times with 0.1% SDS at 65°C for 10 minutes and validated by background signal detection. Volume signal intensities (VSI) were measured using ImageQuant TL 1D v8.1 (GE Healthcare, Chicago, MA, USA). A standardized template was designed and used for equal dot area measurement of all blotted samples. C and G circle quantity were calculated in arbitrary units (AU) after background correction and normalization to alu‐signal as described.^19^ All results were divided by 3 500 000 to make VSI numbers easier to handle. Then VSI of global background control obtained from samples with omitted DNA was subtracted. This was followed by correction for sample‐specific background due to non‐polymerase products. In detail, for each DNA sample the global background corrected VSI value from CCA reaction without polymerase (phi‐) was subtracted from the corresponding value of CCA reaction with polymerase (phi+). Finally, correction for the DNA amount per CCA reaction performed was calculated by dividing global background corrected numbers of CCA reaction products by global background corrected numbers of Alu element repeats. CCA levels were given in arbitrary units (AU). Following published recommendations, AU of more than 5‐fold above background (>1.18 AU) indicate ALT activity and AU less than 2‐fold above background are considered as ALT‐negative, because they are indistinguishable from background noise.^19^ Levels that are between two‐ to 5‐fold the background levels need to be carefully assessed with the appropriate controls.

Total telomeric content (TC) of all samples was calculated by using the so‐called Telo/Alu method by dot blot analysis with radiolabeled gtel oligonucleotide.^36^, ^37^ In brief, telomeric dot blot VSI of phi‐ CCA reactions were normalized to corresponding values of Alu element repeats obtained from rehybridization experiment after stripping the membrane and after background correction as described above.

### 2.5 Statistical analysis

Statistical analysis was performed using the GraphPadPrism 5 software package (Version 5.02). Correlation was determined by Spearman correlation coefficient and paired *t* test to compare results from CCA. The statistical analysis of clinical data was performed with the statistical software package SPSS v.25 for Windows (IBM SPSS, IBM Corporation, Chicago, USA). ALT status was correlated with the factors sex, breed (Rottweiler against others) and tumour location (tibia against another site) by a two‐tailed Fisher's exact test. The Kaplan–Meier product limit method was used to estimate median OST. For calculation of OST, all deaths were included. Differences between Kaplan‐Meier curves were assessed by the log‐rank test. The potential influence of ALT status and other historical prognostic factors on OST was evaluated by Cox proportional hazard model. As historical prognostic factors young age (< 5 years), location (humerus vs other), metastasis at diagnosis and ALP‐elevation were used.

## 3 RESULTS

### 3.1 C‐Circles detected in canine osteosarcomas above ALT threshold

**FIGURE 1:**
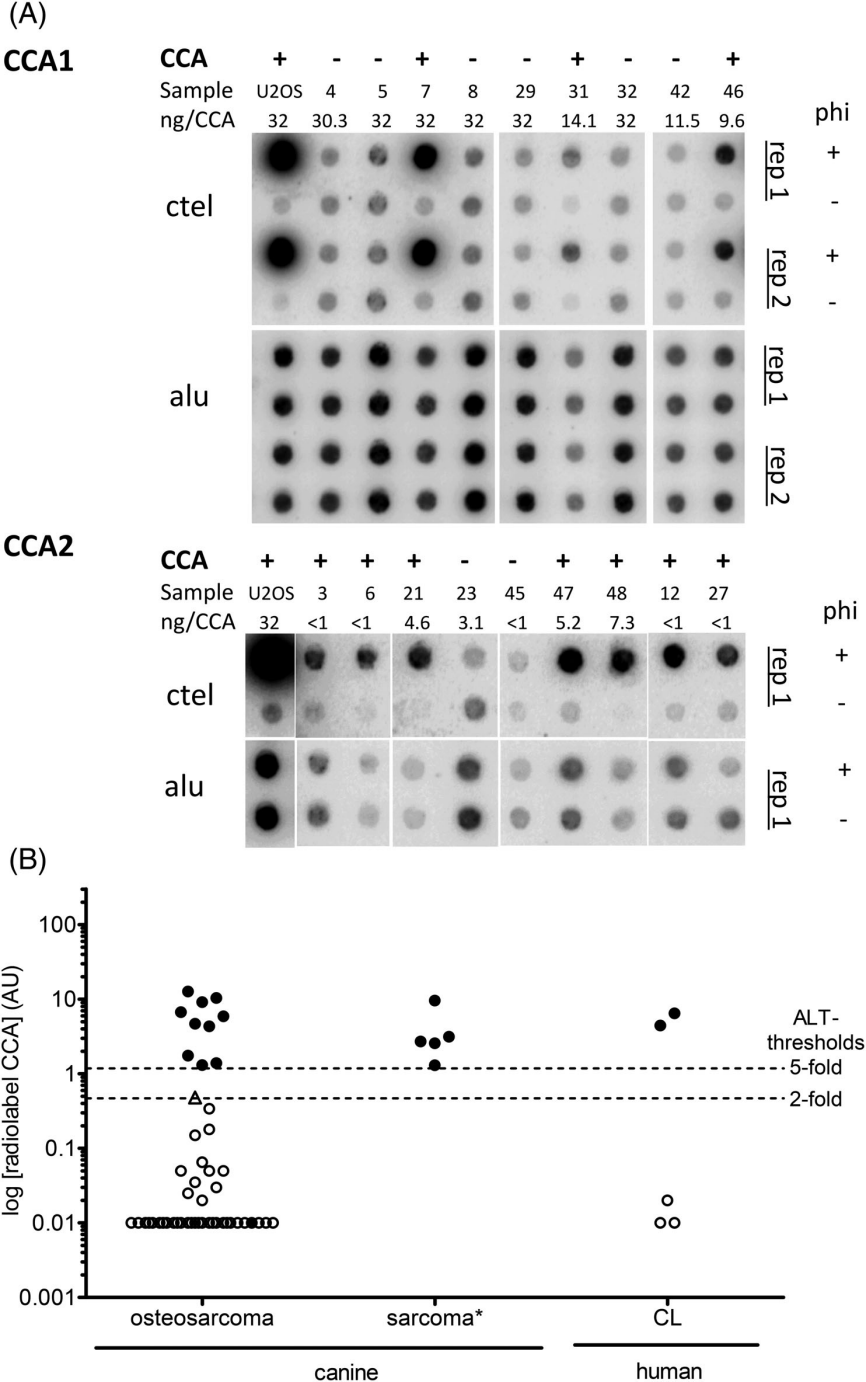
Radiolabel C‐circle assay (CCA) of 50 canine osteosarcoma samples. A, Results of two dot blots (CCA1 and CCA2) including the 10 CCA positive (CCA+), representative CCA negative (CCA−) osteosarcoma samples and the human ALT+ cell line U2OS as positive control hybridized with ctel and alu oligonucleotides are shown. Input DNA per sample is displayed in the row ng/CCA. Samples of CCA1 were analysed in two technical replicates (rep 1 and rep 2). B, ALT activity levels in arbitrary units (AU). The 10 CCA+ osteosarcoma samples, known CCA+ canine FFPE sarcoma (*) samples and human ALT control cell lines (CL) show CC levels above the human 5‐fold positivity threshold (= 1.18 AU) and are marked by full dots. Samples below 2‐fold above background (= 0.48 AU) were considered CCA− (open symbols), including one sample with an AU level of borderline 2‐fold above background (triangle). Samples were analysed according to the detailed method published.^19^ The dashed lines indicate thresholds of CCA levels 2‐ and 5‐fold above background

Archived FFPE tumour tissue samples from 50 canine osteosarcoma patients were assessed for ALT activity using the radiolabel C‐circle assay (CCA) (Table 1). The CCA detected elevated CC levels above the published 5‐fold background threshold in 10 (20%) cases (Figure 1). Cases were grouped as CCA+ or CCA‐ according positive or negative CCA results (Table 1).

**TABLE 1:** Demographic and clinical characteristics of 50 dogs with appendicular osteosarcoma

Parameter	Cases with data (%)	CCA+	CCA−
Sex	50		
Male	22 (44)	8	14
Female	28 (56)	2	26
Neuter status	24		
Male castrated	9 (18)	3	6
Female spayed	15 (30)	1	14
Age categories	50		
<5 years	4 (8)	0	4
5–10 years	37 (74)	9	28
>10 years	9 (18)	1	8
Size categories	50		
Giant (>60 kg)	6 (12)	2	4
Large (30‐60 kg)	31 (62)	6	25
Small/medium (< 30 kg)	13 (26)	2	11
Breed	50		
Purebred	36 (72)	9	27
Mix breed	14 (28)	1	13
Common Purebred	50		
Rottweiler	5 (10)	3	2
Bullmastiff	3 (6)	1	2
Doberman	3 (6)	0	3
Sample collection	50		
Amputation	21 (42)	5	16
Core biopsy	29 (58)	5	24
Common tumour site	50		
Humerus	20 (40)	2	18
Tibia	11 (22)	4	7
Femur	7 (14)	0	7
Ulna	5 (10)	1	4
Common tumour subtype	33		
Osteoblastic	15 (30)	8	7
Fibroblastic	6 (12)	1	5
Low differentiated	3 (6)	1	2
Alkaline Phosphatase	41		
Elevated	15 (30)	2	13
Normal	26 (52)	8	18
Metastasis at diagnosis	50		
No metastasis	46 (92)	9	37
Systemic	3 (6)	0	3
Local	1 (2)	1	0

Input DNA content for the CCA ranged between 1 and 32 ng in 42 samples. In 8 of 50 samples, only low input DNA with less than 1 ng was available for the CCA. Four of the ten CCA+ cases were identified within these eight samples (Figure 1A). Input DNA content did not correlate with the AU level, ruling out a dependency between these two parameters (Spearman r − 0.041, *P* = .7799). On the other hand, input DNA content and measured numbers of Alu element repeats correlated with each other (Spearman r 0.7723, *P* < .0001). DNA Alu signals of corresponding phi + and phi− dots as well as duplicates showed good agreement, ruling out signal intensity variations due to inaccuracy during the assay execution process.

CCA+ and CCA− samples could be well separated (Figure 1A, B). All CCA+ samples showed stronger signals with addition of Phi29 DNA polymerase (phi+) allowing rolling circle amplification of CC than without enzyme addition (phi−). In CCA− samples, signals with polymerase (phi+) were similar to sample‐specific background signals without polymerase (phi−). In the ten CCA+ cases CC signals were all higher than the 5‐fold above background threshold (1.18 AU) and ranged from 1.4 to 12.8 AU (median level = 4.7 AU) (Figure 1B). The AU levels in CCA− samples ranged from −0.13 to 0.41 AU (median level = −0.01 AU). One sample reached the 2‐fold above background level (0.48 AU) and was classified as CCA−. For 20 (33%) cases the samples from technical replicates were analysed by CCA (Figure 1B) and AU levels of replicates correlated very well (R = 0.9789; *P* < .0001). ALT activity was confirmed in positive controls from canine sarcoma tumour tissue samples and two human ALT cell lines. In the canine sarcoma controls AU levels ranged from 1.3 to 9.6 AU (median level = 2.65 AU) and the human cell lines had AU levels of 4.4 AU for U2OS and 6.5 AU for YTBO. The three human ALT negative control cell lines with telomerase activity showed AU levels between 0.00 and 0.02 AU (Figure 1B).

In summary, ALT activity was detected in 20% of canine appendicular osteosarcoma FFPE tumour tissue samples as well as known canine and human positive controls by radiolabel CCA.

### 3.2 G‐circles and telomeric content in canine osteosarcomas

In humans G‐circles (GCs) are considered to be ALT specific although about 100‐fold less abundant compared to CCs.^15^, ^16^ Therefore, we also measured GC levels in comparison to CC levels in the canine osteosarcoma samples. GC levels were about 20‐fold lower than CC levels. GC levels of CCA+ cases ranged from −0.25 to 0.01 AU (median level = 0.005 AU) without correlation between CC and GC levels (Spearman r = 0.431, *P* = .218, data not shown). CCA− samples had GC levels between −1.25 and 0.19 (median = 0.004 AU). In canine ALT controls GC levels ranged from −0.01 to 0.47 AU (median = 0 AU). Human ALT control cell lines showed GC levels of 0.6 AU for U2OS and 0.01 AU for YT‐BO. GC levels in human telomerase controls ranged between 0.000 and 0.001 AU.

**FIGURE 2:**
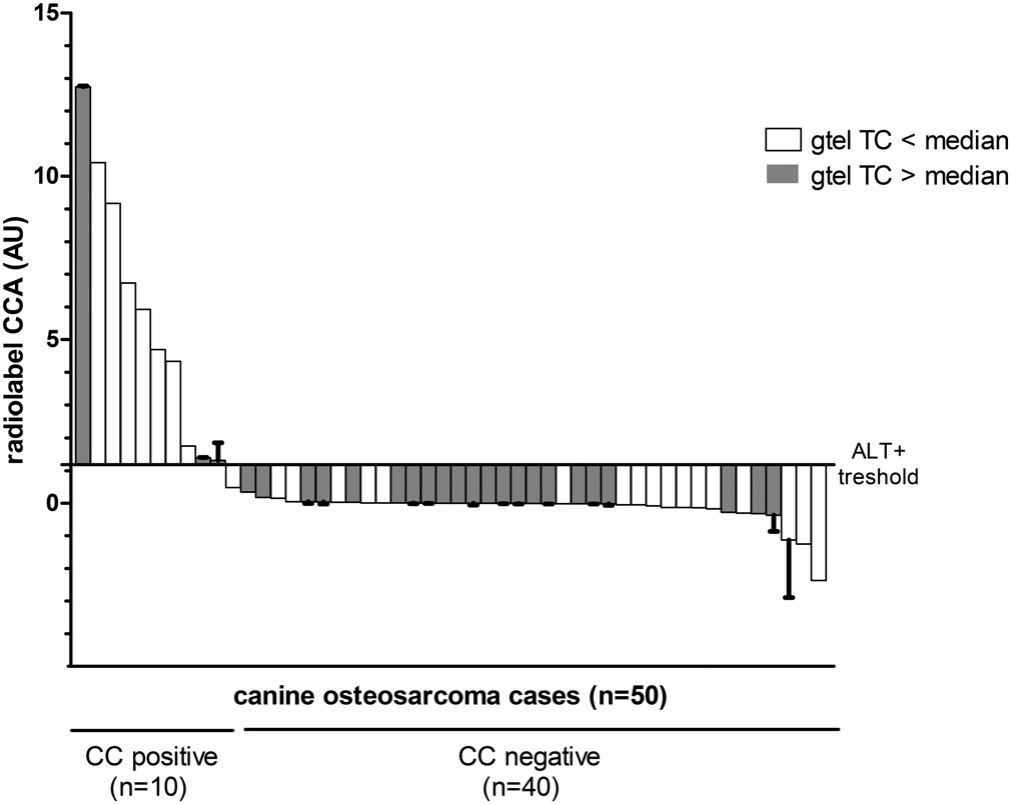
Waterfall plot of CCA results compared to median radiolabel TC. Bars show CC levels of the 50 canine osteosarcoma samples in arbitrary units (AU) with the classified 10 ALT positive samples above and 40 ALT negative‐samples below the baseline. Baseline indicates the human 5‐fold ALT− positivity threshold (= 1.18 AU). SD of replicate samples (n = 20) are shown. Filled bars indicate samples with a radiolabel gtel TC above median (> 0.06) whereas open bars indicate gtel TC below median

Measurements of total telomeric content (TC) by radiolabel Telo/Alu method of canine osteosarcoma samples ranged from −0.02 to 1.16 AU. Median TC level of all samples analysed was 0.06 AU, of CCA− samples 0.08 AU and of CCA+ samples 0.02 AU. Only 3 of the 10 CCA+ cases classified as ALT showed TC values above median (1.06 to 1.16) (Figure 2).

No correlation between these two parameters CCA+ and TC were found (Spearman r = 0.114, *P* = .4307). TC of canine sarcoma tumour tissue controls with known ALT activity varied between 0.02 and 1.02 AU. The two human ALT controls had a TC level of 1.04 for U2OS and a low TC of 0.005 AU for YT‐BO. The three human telomerase controls had a low TC ranging between 0.0015 and 0.0036 AU.

Similar as in human, in canine osteosarcoma the measurements of GC level and TC by telo/alu method are not suitable for detection of ALT activity.

### 3.3 ALT defined by CCA‐status and associations with clinical characteristics

Demographic patient characteristics of the 50 dogs included into the study were grouped as ALT+ and ALT− according the CCA‐status (Table 1).

Mean age of all 50 patients at the time of diagnosis was 8 years. There was no difference in mean age at diagnosis between ALT+ and ALT− patients. The majority of dogs were middle‐aged and older dogs (92%) as well as large or giant breed dogs (74%). Male dogs were overrepresented in the group of CCA+ patients with 8/10 dogs and there was a significant association between CCA− status and sex by 2‐tailed Fisher exact test (*P* = .014). In the CCA+ patient group nine of ten patients were purebred dogs and the Rottweiler was the most common breed. There was significant association between CCA− status and breed (Rottweiler vs others) by two‐tailed Fisher exact test (*P* = .048**)**. The only mix breed dog in the CCA+ group was a Rottweiler‐mix. Other breeds diagnosed with an CCA+ OSA were Bullmastiff, Anatolian Shepherd, Landseer, Boxer, Large Munsterlander and Dachshound (each n = 1). In the CCA+ patients, the proximal tibia was the most common site (4/10), but there was no association between CCA‐status and location (tibia against others). Tumour subtype was recorded in 33/50 specimens. The most frequently recorded tumour subtype in the whole cohort (n = 15) as well as the CCA+ subgroup (n = 8) was osteoblastic osteosarcoma. Elevated pretreatment serum ALP level was observed in 15 dogs (30%), including two CCA+ patients. In 46 dogs, no systemic or local metastases were present at the time of diagnosis. In three CCA− dogs beginning lung metastasis and in one CCA+ dog local lymph node metastasis was reported.

An oncologic treatment was administered in 24 dogs (48%), whereas 26 dogs (52%) received only symptomatic pain medications without any oncologic therapy. Of the 24 treated patients, 15 dogs received a definitive and nine dogs a palliative therapy. Interestingly, all four dogs with metastasis at diagnosis were in the definitive treatment group. Definitive treatments included gold‐standard amputation and adjuvant chemotherapy in 12 dogs. Chemotherapeutic protocols included single agent carboplatin (270‐300 mg/m^2^), doxorubicin (30 mg/m^2^ or 1 mg/kg if less than 15 kg) or a combination of alternating both drugs. Three dogs received limb salvage with distal ulnectomy, partial metatarsectomy with digital amputation and limb sparing surgery for distal radius. All three patients were treated with adjuvant chemotherapy and the ulna/metatarsus site was further treated post‐surgery with hypofractionated radiotherapy. Palliative treatments included amputation alone, palliative radiotherapy with or without chemotherapy and chemotherapy alone. Seven patients including 3/4 definitively treated dogs with “metastasis at diagnosis” received some form of rescue therapy after first signs of tumour progression (metastatectomy, rescue chemotherapy or palliative radiotherapy). Three of these seven dogs had a CCA+ tumour and four dogs a CCA− tumour. Thirty‐six dogs died of tumour‐related reasons. Three dogs were euthanized due to others causes and 11 patients were lost for follow‐up. Average time of follow‐ up for all patients was 215 days (range 4‐1382).

**FIGURE 3:**
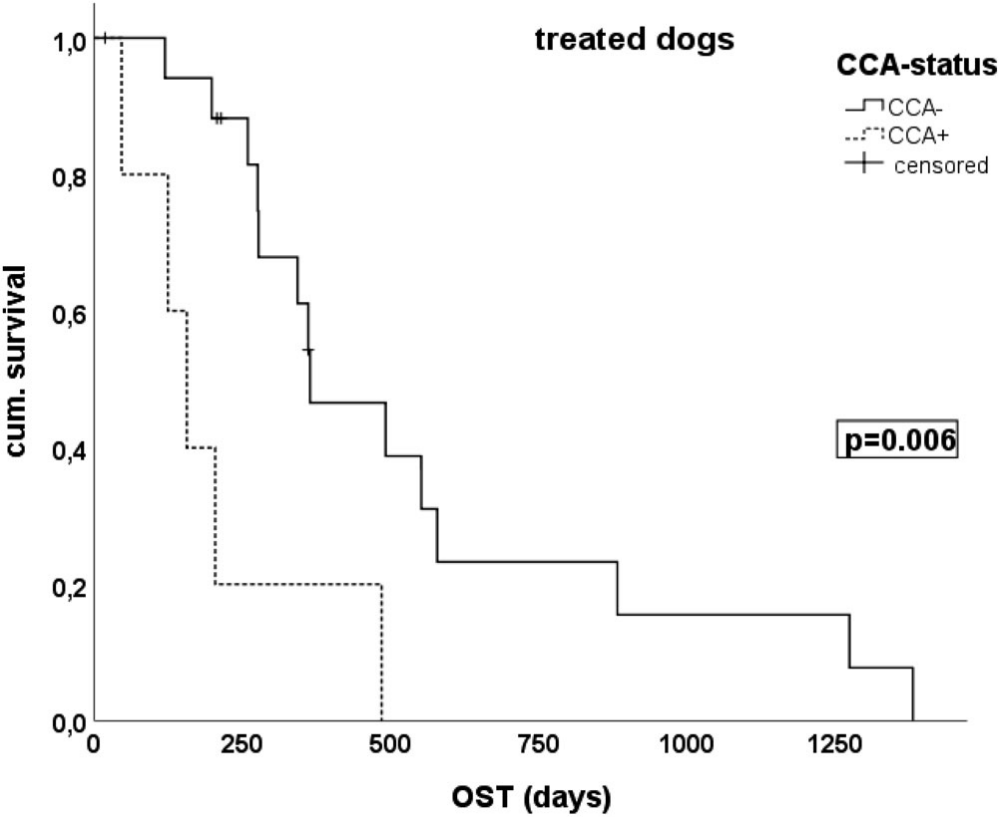
Kaplan–Meier curves of median overall survival (OST) in treated dogs with appendicular osteosarcomas depending on CCA− status. CCA+ patients receiving an oncologic therapy show shorter OST than CCA− patients

Median OST for all 50 dogs was 168 days (range 4‐1382 days). Median OST in the 26 untreated dogs was 45 days and 346 days in 24 treated dogs (*P* < .001). In the group of treated dogs, patients with CCA+ tumours had a median OST of 156 days in comparison to 364 days in CCA− tumours (*P* = .006) (Figure 3). In the untreated group median OST was 16 days in CCA+ dogs in comparison to 58 days in CCA− dogs (*P* = .069) (Table 2). CCA− status remained a significant prognostic factor besides the factor “oncologic therapy” in the Cox proportional hazard model (*P* = .002) (Table 3). None of the historical prognostic factors including “metastasis at diagnosis” had a significant influence on survival in our study (Table 3). The latter finding might be explained by the fact that the few dogs with “metastasis at diagnosis” received aggressive treatment including salvage therapy.

**TABLE 2:** Summary of Kaplan–Meier estimates for overall survival (OST) depending on CCA‐status

Treatment	Cases (n)	Cens. (n)	Median OST (d)	SE	95% CI	Chi‐square	*P*‐value
Untreated							
CCA+	4	0	16	12	0‐39		
CCA−	22	7	58	8	29‐61	3.30	.069
Treated							
CCA+	6	1	156	35	87‐225		
CCA−	18	4	364	60	244‐478	7.57	.006

**TABLE 3:** Cox proportional hazard ratio shows CCA− status as a significant prognostic factor in dogs with appendicular OSA

Factor	*P*‐value	Hazard ratio	
		ExpB‐value	95% CI
Oncologic therapy	.001	0.133	0.055‐0.326
CCA− status	.002	4.043	1.642‐9.953
Young age (< 5 years)	.849	1.132	0.318‐4.026
Humerus location	.581	0.817	0.400‐1.672
Metastasis at diagnosis	.448	0.629	0.190‐2.087
Elevated ALP‐level	.917	1.046	0.447‐2.449

## 4 DISCUSSION

In this study we demonstrated by detection of partially single‐stranded telomeric CCs that the telomerase‐independent telomere maintenance mechanism ALT is active in 20% of canine appendicular osteosarcomas.

The detection of ALT specific CCs by the CCA has developed to a front‐line ALT assay because it is a highly specific and sensitive assay.^16^, ^19^, ^20^ An update of the original radiolabel CCA protocol as recently been published with slight modifications and was used in the present study.^15^, ^19^ In our previous study of 64 various canine sarcomas we already demonstrated that ALT+ human control samples showed comparable CC levels as canine samples and that the published human ALT thresholds were applicable for canine tumour specimens.^14^, ^15^, ^20^ This comparability of ALT activity detection between the species was again confirmed in our present study. Samples with CC levels above the 5‐fold background threshold were scored positive as recommended.^16^, ^20^ We further looked at the 2‐fold background level because this level is described as suspicious for ALT activity.^19^ From the 40 CCA negative samples one sample closely reached this 2‐fold above background threshold. All other CCA negative samples were clearly below the 2‐fold threshold showing a good and clear separation in AU‐values between ALT+ and ALT‐ cases. In eight of 50 samples (16%), only low amounts of DNA were extracted from FFPE tissue and resulted in input DNA below the cut‐off for DNA measurement of less than 1 ng for the CCA. High proportions of tumour matrix with low cellular density in some osteosarcoma tissue sections might have caused the low DNA extraction. The CCA was able to amplify and detect existing CCs in four of these samples. The linearity of the radiolabel CCA has been shown up to at least 32 ng genomic DNA. Factors that are described to affect linearity are high amounts of genomic DNA (> 64 ng) that competes for the polymerase but not low amounts of DNA.^19^ False‐negative ALT activity in four other samples with less than 1 ng DNA applied for CCA could be excluded as comparable alu dot blot signals to the four CCA+ cases were detected. Alu signals report the DNA input applied for the CCA. However, as ALT activity differs 10‐fold within a range of 1.31‐12.74 AU between the identified ALT+ cases, tumours with weak ALT activity signals and less DNA applied for CCA might not be detectable as ALT cases.

G‐circles (GCs) are partially single‐stranded telomeric DNA circles that have as C‐circles the ability to self‐prime RCA of G‐strand templates and are also considered as ALT specific.^15^, ^16^ Because GCs are about 100‐fold less abundant than CCs they have hardly been investigated and are not used as a marker for ALT detection in humans.^16^ In dogs, GCs have not been described yet. Therefore, we evaluated, if GCs were quantifiable in our canine osteosarcoma samples. GC levels were about 20‐fold less abundant than CCs and did not correlate with measured CC levels, probably due to the very low signal intensities of GCs. GC levels were not suitable to separate ALT+ and ALT− canine tumours.

Long heterogeneous telomeres are another ALT characteristic. Measurement of telomeric content (TC) by qPCR CCA as a substitute for the gold standard TRF analysis can support the detection of an ALT phenotype.^20^ Recently, an approach to determine TC by radiolabeled dot blot assay has been described, using gtel and alu hybridization signals for TC calculation.^36^, ^37^ Telo/Alu dot blot signals were proportional to telomere length measured by TRF in human cell lines.^37^ The method was applied to canine FFPE tumour tissue samples in this study. No association between Telo/Alu dot blot signals and presence of CCs was found and this method was not suitable to distinguish canine CCA+ and CCA− cases. This result is in agreement with a human study where the Telo/Alu method missed cases with long telomeres (16/34) and measured an increased Telo/Alu signal despite short telomeres by TRF in 24/91 tumour samples.^36^ Our finding is also consistent with our previous study in which TC measured by the qPCR CCA method was not a reliable tool to distinguish ALT+ and ALT− tumours.^14^ Possible explanations are that the ALT phenotype is known to generate heterogeneous short and long telomeres.^38^ However, the measured telomere content (TC) of bulk cells may not show these individual longer telomeres but instead report the mean telomere length of all present cells. Some CCA+ tumours might use telomerase as second TMM as described for human osteosarcomas and this might result in similar TC levels as for CCA− cases.^27^, ^29^ Further, chromosomal instability with chaotic karyotypes and telomere dysfunction described in canine and human osteosarcomas can explain why TC is not a reliable tool to identify ALT.^39^, ^40^ Further, in dogs telomere length in tumour and normal tissue has more variability than in humans with a strong overlap in telomere length and even identical mean TRF length, making TC less suitable to identify ALT.^41^ Our finding is however consistent with the human literature, where telomere length alone is also not suitable for ALT detection.^20^, ^42^

Patient characteristics of our canine study cohort were consistent with a recent report on demographic characteristics and phylogenetic distribution of 744 dogs with appendicular osteosarcomas and other previous studies.^31^, ^43^, ^44^ In the group of CCA+ patients, male dogs were overrepresented with a significant association between CCA− status and male gender. Several clinical studies in canine osteosarcomas report a slight overrepresentation of male dogs independent from any knowledge about TMM status.^30^, ^45^ No association of ALT phenotype with male gender has been reported in human osteosarcomas. Our finding needs further investigations in a larger patient cohort. Mean age at diagnosis was not significantly different between CCA+ and CCA− dogs. This is in concordance with some human reports.^27^ Other human studies observed that ALT+ osteosarcoma patients were younger than ALT− patients and no ALT activity was found in patients older than 45 years.^26^ In our cohort, no dog with an CCA+ osteosarcoma was older than 11 years. It can be speculated, that ALT phenotype occurs less frequently in senior patients in both species. In canine osteosarcoma, age‐distribution is influenced by phylogenetic breed cluster.^31^ This fact together with our observation that 9/10 dogs affected from CCA+ osteosarcomas were purebred dogs, suggests a contribution of genetic and possible inherited epigenetic factors in ALT activation.^17^, ^35^ Rottweiler was the most common purebred dog in our CCA+ group with a significant association between ALT status and this breed. The only mixbreed dog in the CCA+ group was a Rottweiler‐mix. Further studies are needed to answer, if the Rottweiler has a genetic predisposition for ALT+ osteosarcomas. If certain dog breeds have a higher prevalence for the ALT phenotype, these breeds would be of special interest as model for comparative ALT research in humans.

The detection of an ALT phenotype in 20% of our study population is consistent with the findings of our initial study in 64 various sarcomas, in which ALT was detected in 25% of four osteosarcoma samples.^14^ It is also in agreement with an earlier study that investigated telomerase activity (TA) in 67 canine osteosarcomas.^10^ TA was detected in 73% and no TA was found in 27% of the dogs. TRF analysis was performed in 10 TA− samples and 9/10 displayed a heterogeneous telomere length suspicious for an ALT phenotype. No specific ALT assay was performed at that early time. The finding that a significant proportion of canine appendicular osteosarcomas use ALT as TMM is relevant for comparative TMM research. Human osteosarcomas utilize ALT as their preferred TMM with a high prevalence rate of approximately 64%.^18^, ^46^ In 14% to 27% of human osteosarcomas, ALT activity occurs together with telomerase activity,^27^, ^29^ instances not occurring regularly in tumours.^9^ For ALT+/TA+ tumours, a mosaic of two mixed tumour cell populations using either telomerase or ALT have been described and in vitro studies have further shown that both TMM can coexist within the same cells.^47^, ^48^ It is currently unknown, if ALT activity in these tumours is not utilized as “true” TMM but is epiphenomenon of transformation. Genetic instability of osteosarcomas may render TMMs moot and it is known that TMM activation is a late event in human osteosarcomas with up to 19% without any detectable TMM.^27^, ^29^ From our study, we cannot answer, if telomerase and ALT may co‐exist within canine osteosarcomas to utilize both TMMs, and this question is interesting for future investigations. The majority of our canine osteosarcomas studied most likely used TA and some might lack both TMMs altogether as described in previous studies.^10^, ^14^ Such tumours with lack of TMMs may represent a distinct class of cancer with a more favourable outcome.^9^, ^27^ In some human sarcoma subtypes ALT phenotype is linked to aggressive biologic behaviour and a negative prognosis.^23^, ^24^, ^26^ In a recent meta‐analysis in soft tissue sarcomas, the risk of mortality was higher for patients with ALT+ tumours.^9^, ^49^ In contrast, for human osteosarcomas the influence of ALT activity on survival is less clear.^26^, ^27^, ^29^ The absence of any TMM has been reported as favourable prognostic factor^27^ and the presence of TA as negative factor.^29^ In both studies ALT status itself was not analysed as prognostic factor. In the latter study, the TA+ group included only 14 tumours and 43% of them had additional ALT activity. It can be speculated that the ALT activity in nearly half of the TA+ osteosarcomas influenced their inferior outcome. Interestingly, only one human study investigated primarily the ALT status as prognostic factor for patient survival.^26^ ALT status was associated with a shorter median survival time, but without statistical significance. Looking at the Kaplan‐Meier survival curve of this small patient cohort (n = 39), many patients were censored and this together with patient number could explain that statistical power was too low. In our study, dogs with ALT activity also had a shorter OST in the group of treated dogs (*P* = .006) and in the group of untreated dogs (*P* = .069) indicating that CCA− status might influence patient outcome. Further, CCA− status was a significant predictor for survival in the Cox proportional hazard model in our study cohort. However, due to the retrospective clinical data collection with only overall survival data and the small heterogeneous patient cohort some unintended treatment bias as well as the bias by time point of euthanasia cannot be excluded in these preliminary results. Therefore, prospective clinical studies with standardized staging, treatment and follow‐up procedures and availability of fresh frozen tissue samples are warranted to further evaluate, if ALT activity can serve as novel prognostic factor in canine osteosarcomas.

In summary, limitations of this study were a small heterogeneous study cohort, the retrospective survival analysis without standardized follow‐ups and missing progression free survival times and the availability of only archived FFPE tissue specimens. Therefore, only gold standard and robust radiolabel CCA was performed for ALT detection. No further ALT characteristics or evaluation of telomerase activity that can only be determined in fresh‐frozen tissue could be analysed.

## 5 CONCLUSIONS

Comparable to human osteosarcomas, a significant number of canine OSA use ALT as TMM. This high prevalence supports the notion of canine osteosarcomas as interesting model for comparative TMM research, especially as the dog is already an accepted spontaneous model for human osteosarcomas. In canine, the CCA− status might serve as a prognostic marker, but further research is needed to further test this hypothesis. Furthermore, canine studies may help to foster the assumed role of ALT to become an attractive tool for diagnosis and a target for therapeutic interventions in human cancer.

## CONFLICT OF INTEREST

The authors declare no potential conflict of interest.

## AUTHOR CONTRIBUTIONS

Conceptualization, Klaus Holzmann and Miriam Kleiter; data curation, Ludmila Bicanova; formal analysis, Miriam Kleiter; investigation, Ludmila Bicanova and Theresa Kreilmeier‐Berger; methodology, Theresa Kreilmeier‐Berger and Klaus Holzmann; resources, Martin Reifinger; supervision, Klaus Holzmann and Miriam Kleiter; validation, Theresa Kreilmeier‐Berger, Martin Reifinger and Klaus Holzmann; writing‐original draft, Ludmila Bicanova; writing‐review and editing, Theresa Kreilmeier‐Berger, Martin Reifinger, Klaus Holzmann and Miriam Kleiter. All authors have read and agreed to the published version of the manuscript.

## Data Availability

All data generated or analysed during this study are included in this published article (and its supplementary information files).
